# Isolation and Molecular Characteristics of a CIAV Isolate From Pigeons, China

**DOI:** 10.3389/fvets.2021.669154

**Published:** 2021-08-11

**Authors:** Hongxia Shao, Jinzhi Li, Huisha Yuan, Lifei Ji, Jun Zhang, Wenjie Jin, Kun Qian, Jianqiang Ye, Aijian Qin

**Affiliations:** ^1^Key Laboratory of Jiangsu Preventive Veterinary Medicine, Key Laboratory for Avian Preventive Medicine, Ministry of Education, College of Veterinary Medicine, Yangzhou University, Yangzhou, China; ^2^Jiangsu Co-innovation Center for Prevention and Control of Important Animal Infectious Diseases and Zoonoses, Yangzhou, China; ^3^Joint International Research Laboratory of Agriculture and Agri-Product Safety, the Ministry of Education of China, Yangzhou University, Yangzhou, China; ^4^Institutes of Agricultural Science and Technology Development, Yangzhou University, Yangzhou, China

**Keywords:** CIAV, pigeon, isolation, identification, molecular characteristics

## Abstract

Chicken infectious anemia virus (CIAV) mainly infects chickens and causes immunosuppression. In this study, a CIAV isolate, designated as Pigeon-CIAV-1906, was efficiently isolated from two sick pigeons by inoculating the samples into MSB1 cells. The genome of Pigeon-CIAV-1906 was amplified by PCR and analyzed. The genome size of Pigeon-CIAV-1906 was 2,298 bp with the highest homology (99.5%) to Jilin strain (JL14023) and the lowest homology (91.5%) to Brazil strain (KY024579), which phylogenetically clustered into Group A. Notably, several amino acids such as 139K and 394Q related with high virulence were found in the VP1 of Pigeon-CIAV-1906. The isolation of Pigeon-CIAV-1906 and its molecular characteristics provide evidence for the cross-transmission of CIAV from chicken to pigeon and give novel insights into the molecular epidemiology of CIAV.

## Introduction

Chicken infectious anemia (CIA) is a disease caused by the chicken infectious anemia virus (CIAV), which is characterized by aplastic anemia and lymphoatrophy in the infected chickens (1). CIAV infects chickens through both vertical and horizontal transmission. CIAV infection can affect the coordination of the entire immune regulatory system, and cause immunosuppression and secondary infection with other pathogens (2, 3). CIAV belongs to the genus *Gyrovirus* of the family *Anelloviridae*, which has a single negative circular DNA genome with a size of about 2.3 kb (4, 5). The single strand of the genome of CIAV contains three overlapped ORFs, encoding three viral proteins VP1 (51.6 kDa), VP2 (24.0 kDa), and VP3 (13.6 kDa), respectively (6). VP1 is the capsid protein with neutralizing epitopes (7). VP2 has dual-specifificity protein phosphatase activity and involves in viral assembly (8, 9). VP3, also known as apoptin, can induce apoptosis of chicken thymocytes and plays critical roles in the pathogenesis (10). Chicken is the natural host of CIAV. Although the serological data indicates that pigeon, quail and crow can also be as the hosts for CIAV (11), the isolation of CIAV from these hosts has not been reported. More recently, the identification of CIAV in human feces, blood and skin, and in dog and cat feces was reported (12–14). In this study, a CIAV isolate, designated as Pigeon-CIAV-1906, was efficiently isolated and identified from two diseased pigeons in China.

## Methods

### Clinical Samples

On June 4, 2019, two sick pigeons (No. 1 and No. 2) suspected of the infection of CIAV were sent to our laboratory for diagnosis. The two pigeons had clinical signs of yellow wings, rough feathers and depression and with the atrophy of pale bone marrow.

### PCR Detection

The blood, heart, liver, spleen, kidney, lung, cecum and other tissues of the two pigeons were collected and their genome DNAs were prepared using the DNA extraction kit, and stored at −20°C. For the detection of CIAV, the PCR was performed using the primers CIAV-VP3-F and CIAV-VP3-R listed in Table 1. The size of the PCR amplification was 366 bp.

**Table 1:** Primers for the identification of CIAV.

**Fragment**	**Primers**	**Primer sequence (5^**′**^-3^**′**^)**	**Size**
VP3	CIAV-VP3-F	ATGAACGCTCTCCAAGAAGATAC	366 bp
	CIAV-VP3-R	TTACAGTCTTATACGCCTTTTTGCG	
Fragment 1	CIAV-F-1	GCATTCCGAGTGGTTACTATTCC	843 bp
	CIAV-R-1	CGTCTTGCCATCTTACAGTCTTAT	
Fragment 2	CIAV-F-2	CGAGTACAGGGTAAGCGAGCTAAA	989 bp
	CIAV-R-2	TGCTATTCATGCAGCGGACTT	
Fragment 3	CIAV-F-3	ACGAGCAACAGTACCCTGCTAT	876 bp
	CIAV-R-3	CTGTACATGCTCCACTCGTT	

### Virus Isolation and Identification

The homogenates of the PCR positive samples were inoculated into MDCC-MSB1 cells, and then the cells were incubated at 37°C with 5% CO_2_ in the incubator. The inoculated cells and their supernatants were passaged every 3–4 days. After three passages, the genomes of the inoculated cells and their supernatants were extracted and first detected by PCR using primers CIAV-VP3-F and CIAV-VP3-R. For the amplification of the genome of the isolated CIAV, the three pairs of primers CIAV-F-1/CIAV-R-1, CIAV-F-2/CIAV-R-2, and CIAV-F-3/CIAV-R-3 listed in Table 1 were used.

### Sequence Analysis

The whole genome sequence of the isolate was aligned and compared with the reference strains deposited in Genbank using MEGA6.1 software. The amino acid sequences of VP1, VP2, and VP3 of the isolate were also compared with the reference strains using DNAStar Lasergene software for molecular features.

## Results and Discussion

**Figure 1:**
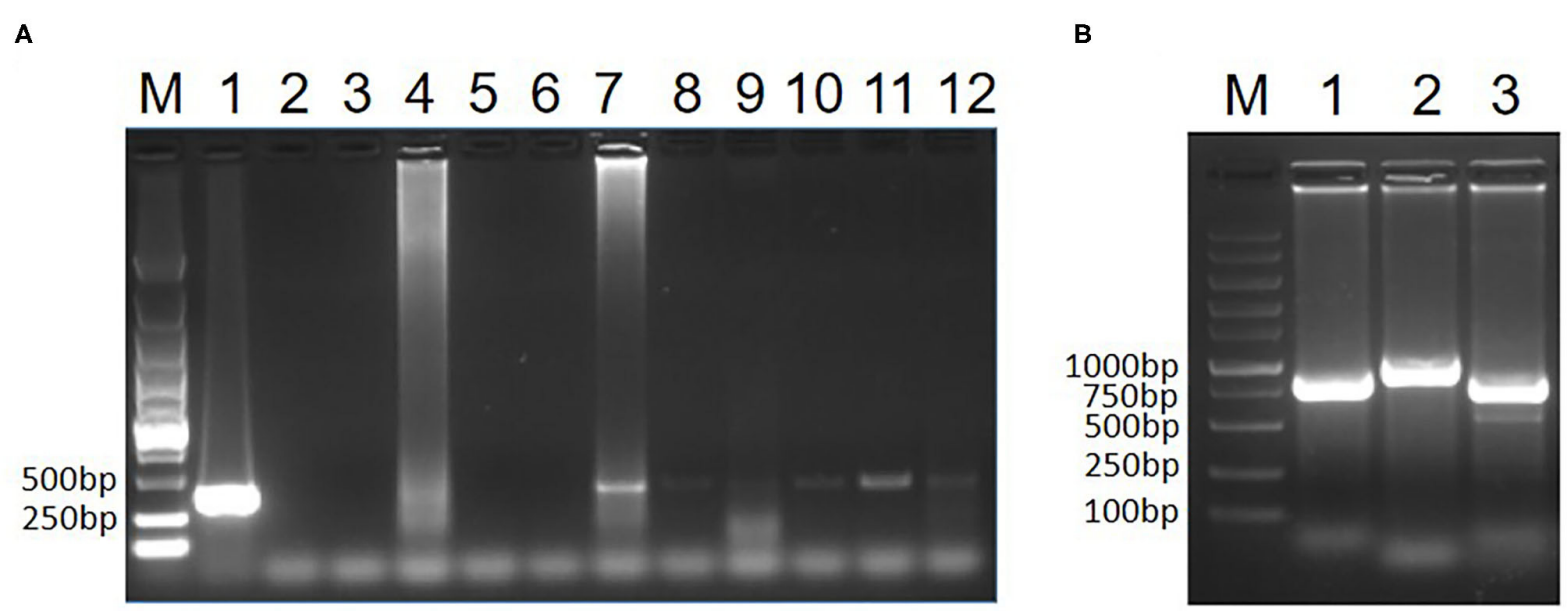
PCR identification of CIAV. PCR detection of CIAV for clinical samples **(A)**. The genome DNA extracted from the two sick pigeons was tested by PCR using primers specific to CIAV. Lane M was a DNA marker; Lane 1 was CIAV positive control; Lane 2 was the negative control, Lane 3, 4, 5, 6, and 7 were the heart, liver, spleen, lung and kidney from pigeon No. 1, respectively; Lane 8, 9, 10, 11, and 12 were the heart, liver, spleen, lung, and kidney from pigeon No. 2, respectively. Whole genome amplification of Pigeon-CIAV-1906 **(B)**. The whole-genome DNA of Pigeon-CIAV-1906 extracted from the supernatant of the infected MSB1 cells was amplified by PCR using primers listed in Table 1. Lane M was the DNA marker; Lane 1, 2, and 3 were the amplified PCR fragment using primers CIAV-F-1/R1, CIAV-F-2/R2, and CIAV-F-3/R3, respectively.

To test whether the two pigeons were infected with CIAV, the genome DNA of the hearts, livers, spleens, lungs and kidneys from the two pigeons was extracted and the PCR for detection of CIAV was performed as previously described (15). The primers for this PCR assay were specific to VP3 of CIAV listed in Table 1. The kidney of pigeon No. 1 and all samples from pigeon No. 2 were positive in the PCR assay for CIAV whereas other samples from pigeon No. 1 were negative (Figure 1). This PCR data indicated that the two sick pigeons were infected with CIAV. To isolate the CIAV, the supernatant from the homogenate of the liver from pigeon No. 2 was inoculated into MDCC-MSB1 cells and the inoculated cells were blindly passaged. Some inoculated cells were swollen and round, and some carried with inclusion bodies compared with the control cells. After three passages, the DNA of the supernatant from the inoculated cells was extracted and the PCR for detection of CIAV was performed. PCR assay showed that a specific band with the size of about 366 bp could efficiently be amplified, highlighting the isolation of a CIAV strain from the pigeon. The isolated pigeon origin CIAV was then designated as Pigeon-CIAV-1906.

**Figure 2:**
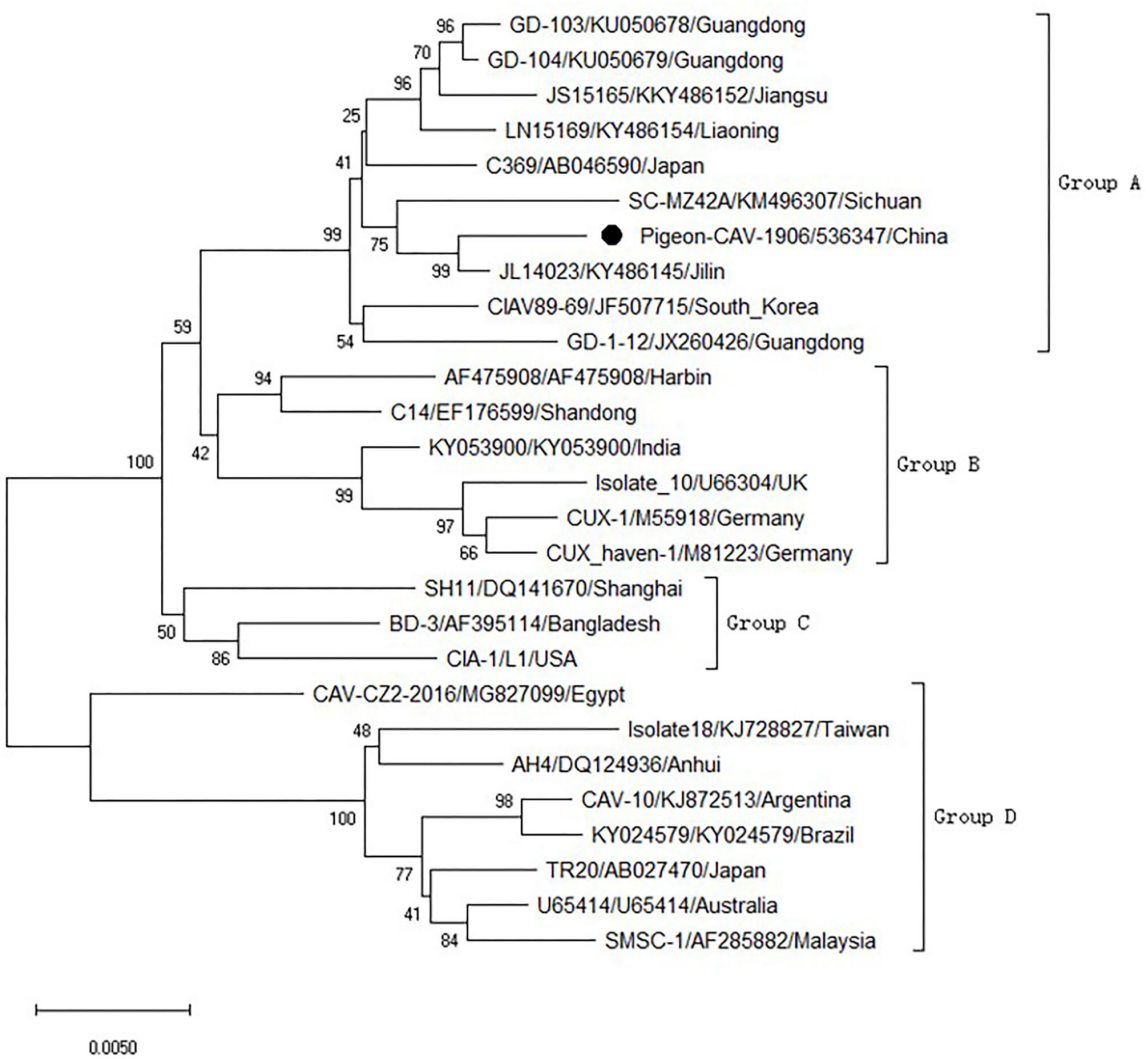
Phylogenetic tree for the genome of Pigeon-CIAV-1906. The phylogenetic tree was generated using the neighbor-joining method (1,000 bootstraps) with MEGA6.

To analyze the molecular characteristics of the isolated Pigeon-CIAV-1906, the complete genome sequence of Pigeon-CIAV-1906 was amplified using the primers listed in Table 1. As shown in Figure 1, three fragments of CIAV with the corresponding size could be amplified by PCR and the genome size of Pigeon-CIAV-1906 was 2,298 bp. The sequence of Pigeon-CIAV-1906 had been submitted to the GenBank (Accession Number: MT536347). The whole genome of Pigeon-CIAV-1906 was compared with that of other 26 CIAV isolates deposited in GenBank, and the genome of Pigeon-CIAV-1906 had 91.5–99.5% homology to that of other CIAV isolates tested. Phylogenetic tree analysis by MEGA6 showed that these CIAV isolates could be divided into four gene groups (Figure 2), named Group A, Group B, Group C, and Group D. Although most of the Chinese CIAV isolates were clustered into Group A, several other Chinese CIAV isolates were also distributed in Group C and Group D, highlighting the diversity of CIAV in China compared with other countries or regions. As showed in Figure 2, Pigeon-CIAV-1906 isolated in this study was clustered into Group A. The pathogenicity and host range of CIAV from different Groups need to be further investigated.

The VP1 from Pigeon-CIAV-1906 and other 495 CIAV isolates collected from NCBI GenBank were analyzed further to study the molecular features in the structural protein. Compared with most sequences, the VP1 of Pigeon-CIAV-1906 carried three mutations including Y13F, L376I, and A413S (Table 2). The three mutations were also found in several other CIAV isolates: in the total 496 isolates tested, 0.4% (2/496), 40.73% (202/496), and 48.99% (243/496) isolates had Y13F, L376I, and A413S in VP1, respectively. For the highly variable region 139-157aa in VP1, like most isolates, Pigeon-CIAV-1906 had 139KSQAAENWPNCWLPLDNNV157. Notably, Pigeon-CIAV-1906 also carried several known virulent determiners such as 75V, 89T, 125L, 139K, 141Q, and 394Q as described in Table 2, indicating that isolate Pigeon-CIAV-1906 is highly pathogenic. Except for VP1, the VP2, and VP3 of Pigeon-CIAV-1906 were also compared with those of other CIAV isolates. Compared with most CIAV isolates, the VP2 of Pigeon-CIAV-1906 carried two mutations including R114Q and T171S (Table 2). In the total 452 isolates tested, 0.88% (4/452) and 0.22% (1/452) isolates had R114Q and T171S in VP2, respectively. The VP3 of Pigeon-CIAV-1906 had a D79N mutation when compared with most CIAV isolates (Table 2). In the total 491 CIAV isolates tested, 1.02% (5/491) isolates carried D79N.

**Table 2:** Mutations and known virulent determiners in Pigeon-CIAV-1906.

**Protein**	**Mutations** ^**a**^	**Known virulent determiners**
VP1	Y13F (2/496 = 0.4%^b^) L376I (202/496 = 40.73%) A431S (243/496 = 48.99%)	75V, 89T, 125L, 139K, 141Q, 394Q
VP2	R114Q (4/452 = 0.88%) T171S (1/452 = 0.22%)	/
VP3	D79N (5/491 = 1.02%)	/

a *The mutation site in Pigeon-CIAV-1906 compared with most CIAV isolates tested*;

b *the percent of this mutation site in the CIAV isolates tested*.

In summary, this is the first demonstration of the isolation and identification of a CIAV (Pigeon-CIAV-1906) from pigeon in China. The isolated Pigeon-CIAV-1906 provides strong evidence that pigeon can be as a host for CIAV infection. Of course, the experimental infection study with Pigeon-CIAV-1906 in pigeons need to be performed to further investigate the pathogenesis and transmission of CIAV in pigeons. The high identity of the genome of Pigeon-CIAV-1906 to that of the CIAV isolated from chickens highlights the cross-transmission of CIAV from chicken to pigeon. Although the functions of the unique mutations in Pigeon-CIAV-1906 compared with most CIAV isolates need to be further investigated, the presence of several known virulent determiners in VP1 of Pigeon-CIAV-1906 indicates the high pathogenicity of the isolated Pigeon-CIAV-1906. Notably, the VP1 of Pigeon-CIAV-1906 carried 394Q, which was reported to be as a critical virulence determinant of CIAV (16–18). Different from chickens, pigeons infected with CIAV have more risks for CIAV transmitting to other wild birds. Therefore, it is critical to perform the epidemiological analysis of CIAV in pigeons for better controlling CIAV in chickens.

## Data Availability Statement

The datasets presented in this study can be found in online repositories. The names of the repository/repositories and accession number(s) can be found at: https://www.ncbi.nlm.nih.gov/genbank/, MT536347.

## Ethics Statement

The animal study was reviewed and approved by The Animal Care Committee of Yangzhou University.

## Author Contributions

HS and AQ conceived and designed the experiments. JL, HY, JZ, and LJ performed the experiments. JL, AQ, and HS analyzed the data. WJ and KQ contributed reagents, materials, and analysis tools. HS, JL, and JY contributed to the writing of the manuscript. HS and JY prepared the figures. All authors read and approved the final manuscript.

## Conflict of Interest

The authors declare that the research was conducted in the absence of any commercial or financial relationships that could be construed as a potential conflict of interest.

## Publisher's Note

All claims expressed in this article are solely those of the authors and do not necessarily represent those of their affiliated organizations, or those of the publisher, the editors and the reviewers. Any product that may be evaluated in this article, or claim that may be made by its manufacturer, is not guaranteed or endorsed by the publisher.
